# Predictors of Headaches and Quality of Life in Women with Ophthalmologically Resolved Idiopathic Intracranial Hypertension

**DOI:** 10.3390/jcm13133971

**Published:** 2024-07-07

**Authors:** Anat Horev, Sapir Aharoni-Bar, Mark Katson, Erez Tsumi, Tamir Regev, Yair Zlotnik, Ron Biederko, Gal Ifergane, Ilan Shelef, Tal Eliav, Gal Ben-Arie, Asaf Honig

**Affiliations:** 1 Department of Neurology, Soroka University Medical Center, Beer Sheva 8453227, Israel; anathorev@clalit.org.il (A.H.); yairzl@clalit.org.il (Y.Z.); galif@clalit.org.il (G.I.); 2 Faculty of Health Sciences, Ben Gurion University of the Negev, Beer Sheva 8453227, Israel; ilans@clalit.org.il (I.S.); galbe@clalit.org.il (G.B.-A.); 3 Ben-Gurion Medical School, Soroka University Medical Center, Beer Sheva 8453227, Israel; sapira.bc@gmail.com; 4 Rambam Health Care Campus, Haifa 3109601, Israel; m_katson@rmc.gov.il; 5 Department of Ophthalmology, Soroka University Medical Center, Beer Sheva 8453227, Israel; erezts@clalit.org.il (E.T.); tamirre@clalit.org.il (T.R.); 6 Clinical Research Center, Soroka University Medical Center, Beer Sheva 8453227, Israel; rbiederko@gmail.com; 7 Department of Radiology, Soroka University Medical Center, Beer Sheva 8453227, Israel; 8 Department of Internal Medicine, Jefferson Einstein Philadelphia Hospital, Philadelphia, PA 19141, USA; talzus@post.bgu.ac.il

**Keywords:** carbonic anhydrase inhibitors, headache, idiopathic intracranial hypertension, transverse sinuses, quality of life, questionnaire

## Abstract

**Background/objectives:** The aim of this study was to evaluate the long-term outcomes of a cohort of ophthalmologically resolved female idiopathic intracranial hypertension (IIH) patients. **Methods:** Our cross-sectional study included adult females with at least 6 months of ophthalmologically resolved IIH. Patients with papilledema or who underwent IIH-targeted surgical intervention were excluded. Participants completed a questionnaire consisting of medical information, the Migraine Disability Assessment Scale (MIDAS) and the Headache Impact Test (HIT-6). Electronic medical records and the results of imaging upon diagnosis were retrospectively reviewed. **Results**: One-hundred-and-four participants (mean age 35.5 ± 11.9 years) were included (7.85 ± 7 years post-IIH diagnosis). Patients with moderate–severe disability according to the MIDAS scale (n = 68, 65.4%) were younger (32.4 ± 8.9 vs. 41.5 ± 14.4 year-old, *p* < 0.001), had a shorter time interval from IIH diagnosis (5.9 ± 5.3 vs. 11.7 ± 8.5 years, *p* < 0.001), and had lower FARB scores (indicating a more narrowed transverse-sigmoid junction; 1.28 ± 1.82 vs. 2.47 ± 2.3, *p* = 0.02) in comparison to patients with low–mild disability scores. In multivariate analysis, a lower FARB score (OR 1.28, 95% CI 0.89–1.75, *p* = 0.12) and younger age (OR 1.09, 95% CI 0.98–1.19, *p* = 0.13) showed a trend toward an association with a moderate–severe MIDAS score. Moreover, in the sub-analysis of patients with a moderate–severe MIDAS scale score, the 10 patients with the highest MIDAS scores had a low FARB score (1.6 ± 1.1 vs. 2.7 ± 2.4, *p* = 0.041). **Conclusions:** High numbers of patients with ophthalmologically resolved IIH continue to suffer from related symptoms. Symptoms may be associated with the length of time from the diagnosis of IIH and a lower FARB score.

## 1. Introduction

Idiopathic intracranial hypertension (IIH), also known as pseudotumor cerebri, is a neurological disorder characterized by elevated intracranial pressure (ICP) with no apparent cause. The underlying etiology of IIH is increased pressure in the dural venous system, but several factors may play a role in the condition’s pathogenesis. IIH predominantly affects young, obese women during their childbearing years. Diagnosis is based on papilledema, normal cerebrospinal fluid analysis, and increased opening pressure on lumbar puncture with no apparent secondary cause of intracranial hypertension [1]. Symptoms may include chronic, severe headaches, transient visual obscurations, pulsatile tinnitus, cognitive dysfunction, and depression. Vision loss is the most concerning symptom as it can lead to blindness [2].

While vision loss in patients with IIH can be evaluated with neuro-ophthalmologic tests, assessing and treating headaches and other IIH-associated symptoms can be more challenging. Moreover, despite their significant impact on daily function and quality of life (QoL), these subjective symptoms are often not considered medically dangerous and so may be overlooked.

The primary goal of IIH treatment is to preserve vision and reduce headaches. Weight loss and medications, primarily carbonic anhydrase inhibitors (CAIs), are first-line treatments for IIH. However, these medications can have a wide range of side effects, with incidence rates as high as 80–100%. Furthermore, more than 10% of patients with IIH are intolerant or nonresponsive to high-dose CAI medical therapy [3,4,5,6,7]. In these cases, surgical procedures such as ventriculoperitoneal shunt, optic nerve fenestration, or transverse sinus stent may be considered [8].

In addition to the potential for severe visual loss and the often-debilitating related symptoms mentioned above, poor QoL has emerged as a key morbidity for patients with IIH [9]. IIH has a significant impact on patient QoL, with headaches identified as the main contributor to reduced QoL. However, treatment with the goal of improving QoL can be difficult, especially in patients who have carried the diagnosis for many years [9,10,11,12,13]. Therefore, this study aims to find predictors of severe headache symptoms and impaired daily function in chronic IIH patients without active ophthalmological involvement.

## 2. Materials and Methods

### 2.1. Research Hypothesis

Our hypothesis was that several structural and physiological changes take place in female IIH patients. As a result, symptoms would improve with a longer duration since diagnosis. In addition, we hypothesized that lower FARB scores (indicating a more narrowed transverse-sigmoid junction) would impair venous drainage and consequently impair normal cerebrospinal fluid (CSF) drainage into the cerebral venous system. As a result, lower FARB scores would be independently associated with a higher degree of IIH symptoms and disability despite longer disease duration because ophthalmological involvement was resolved.

### 2.2. Statement of Ethical Approval and Consent

This study was approved by our medical institutional review board committee [number 0278-2020]. All participants consented to the anonymized use of their clinical and questionnaire data. Informed consent was obtained verbally prior to administering the questionnaire.

### 2.3. Study Population

We included only female IIH patients as most IIH patients are young females, and to reduce possible bias as substantial differences may exist in the underlying pathophysiology of IIH between females and males. Therefore, to be eligible for inclusion, participants had to be female, aged 18 years or older at the time of IIH diagnosis, and have cerebral venous imaging data from their initial IIH diagnosis. In addition, patients were required to have been diagnosed with IIH at least six months ago and to have been treated with CAI for at least three months prior to study enrollment. Finally, patients had to be without any signs of optic disc edema in their most recent ophthalmological evaluation. Pediatric and male patients, or patients who required surgical treatments such as a ventriculoperitoneal shunt, optic nerve fenestration, or cerebral venous stenting, were excluded from the study.

### 2.4. Recruitment and Sampling

Electronic medical records of patients diagnosed in our department with IIH between January 2012 and December 2018 were evaluated. Out of 210 consecutive patients, updated telephone numbers were available for 150 patients, who were called and invited to participate in the study. One-hundred-and-thirty-three patients agreed to participate in the study and complete the questionnaire. After obtaining consent, exclusion criteria were reviewed with the patients based on their electronic medical records. Consequently, 19 patients were excluded either because of the unavailability of cerebral venous imaging data from the time of IIH diagnosis or because they were undergoing a surgical procedure. The remaining 104 patients were enrolled into the study.

### 2.5. Data Collection

Data were collected by five different teams of researchers blinded to each other. All researchers were physicians, and special attention was given during the process of data collection to patients who required changes in their medical care. Retrospective data collection was performed by four different researchers (general medical, neurological, ophthalmological, and radiological) from the patients’ index hospitalization and health medical organization (HMO) electronic medical records. Data collection was performed separately, and every researcher was blinded to the data collected by the other researchers. Additionally, to minimize possible bias, both the questionnaire and radiological review were each performed by a single researcher.

Epidemiological and general medical information including age, height, weight, and body mass index (BMI) on index hospitalization, chronic medical conditions, and medications were collected by a general physician. A neurologist ascertained the diagnosis of IIH using lumbar puncture opening pressure and CSF laboratory analysis on index admission. Ophthalmologists ascertained both the presence of either papilledema or visual field defect upon IIH diagnosis and that all participants were appropriately followed up by a neuro-ophthalmologist. Additionally, they ensured that IIH patients included in the study did not have signs of optic disc edema in their most recent ophthalmological evaluation. The questionnaire was administered within three months following the most recent ophthalmological evaluation. Imaging data were analyzed in a core laboratory by a single radiologist, who ruled out the presence of structural lesions that may contribute to increased ICP, in turn contributing to the diagnosis of IIH. The radiologist evaluated the presence of an empty sella, high jugular bulb, and slit-like ventricles, measured the optic nerve width bilaterally, and determined the dominant side of venous sinus drainage, if available. He graded transverse sinus stenosis (TSS) using the Combined Conduit Score (CCS)—an index introduced in 2003 by FARB et al. [14]. The grading of the patency is performed in each of the transverse sinuses (left and right), and the grade ranges from 0 to 4 based on the level of patency: 0—0% patency, 1—less than 25% patency, 2—25–50% patency, 3—50–75% patency, 4—75–100% patency. The score of each side is then combined to derive the CCS (0–8). A normal healthy result is 8, which signifies no or very little TSS.

### 2.6. Study Questionnaire

All participants enrolled in the study were interviewed between November 2020 and October 2022. The questionnaire consisted of information relating to the symptoms experienced by the participants as well as their general state of health (Supplemental Materials).

The first part of the questionnaire consisted of 35 questions concerning basic demographic characteristics, time since diagnosis, and additional medical history. The second section comprised two existing questionnaires validated for migraine patients: six questions from the Headache Impact Test (HIT-6) [15,16] for the assessment of the impact of headache on daily life and five questions from the Migraine Disability Assessment Scale (MIDAS) [17].

HIT-6 [15,16]: The Headache Impact Test-6 (HIT-6) was developed to measure a wide spectrum of factors contributing to the burden of a headache, and it has demonstrated utility for generating quantitative and pertinent information on the impact of a headache. The disability was classified using the following two impact grades based on the HIT-6 score: little-to-substantial impact (HIT-6 score: 36–59) and severe impact (HIT-6 score: >60) [15,16].

*MIDAS* [17]: The MIDAS is a self-reporting instrument that was administered to patients to measure headache pain intensity and headache attack frequency. Based on the total score, the severity of the migraine is classified into grades I–IV (I = little or no disability, II = mild disability, III = moderate disability, and IV = severe disability) [17].

The third part of the questionnaire focused on patients’ reported outcome measures (PROMs) and included 23 questions. Satisfaction with the current medical treatment and perception of symptoms were assessed using nine questions on a scoring scale ranging from 1 (‘I don’t agree at all’) to 5 (‘Strongly agree’) that included five ‘Yes/No’ questions. Side effects were rated using a single question, with ratings ranging from 0 (‘Not at all’) to 7 (‘Bothers me a lot’). The last eight questions evaluated functional improvement and lifestyle changes since diagnosis (eight ‘Yes/No’ questions and two questions on a 1–5 scale, with ‘1’ being ‘I don’t agree at all’ and ‘5’ being ‘Strongly agree’).

### 2.7. Statistical Analysis

Data analysis was performed using R software version 4.1.0 with R-Studio software version 2022.07.1. Summary statistics are presented as mean and standard deviation (SD) for continuous variables, and as numbers and percentages for binary or categorical variables. The association between every two qualitative variables was tested using the chi-square ($\chi^{2}$) test. Tests between independent samples were performed using an analysis of variance (ANOVA) test after confirming that the data conformed to a normal distribution. A probability value of <0.05 was considered statistically significant, and all tests were two-sided.

The HIT and MIDAS results were analyzed as dichotomous variables. HIT: (1) Little + Mild Severity versus Moderate + Severe Severity, and MIDAS: Little + Mild Severity versus Substantial + Severe Severity (MIDAS > 55). Analysis was also performed according to the median time elapsed since the diagnosis.

Univariate analyses were used to assess differences between the above groups in demographic and clinical characteristics. Overall perception of functional difficulties in daily activities was calculated using a summary of each difficulty (ranging from 0 to 8).

## 3. Results

### 3.1. Demographics and Baseline Clinical Characteristics of Study Participants

One-hundred-and-four participants (mean age 35.5 ± 11.9 years) were diagnosed with IIH an average of 7.85 ± 7.01 years prior to participating in the study (Table 1). Ninety-two patients (88.4%) had papilledema, and 39 (37.5%) had a significant visual field defect (other than enlargement of the blind spot) upon diagnosis. One-hundred-and-four participants answered the PROMs and MIDAS questionnaire while only 102 patients answered the HIT-6 questionnaire.

### 3.2. Headache, Function, and Patient-Reported Outcome Measures (PROMs)

A high proportion of the participants reported significant difficulties in daily activities, such as watching television (31.2%) or reading (37.1%) for a long period of time, driving (25.6%), learning new information (25.5%), remembering where things were placed (38.1%), and remembering whether tasks were completed (40.2%). Furthermore, about one-third (33.9%) of the participants reported no improvement in their general physical condition, 35.8% reported difficulties in completing tasks to the best of their ability, 32% reported difficulties in keeping up with work or studies, and 39% reported a lack of happiness. Additionally, almost half of the participants (47.8%) reported feeling discouraged by the ability of the medication to treat their illness.

### 3.3. Medical Treatment

Twenty-nine patients (27.9%) were treated with acetazolamide or topiramate at the time of the questionnaire’s administration. Of the overall study population, only 73% adhered to the instructed CAI treatment protocol. Sixty-five participants (62%) reported significant adverse effects associated with the usage of CAI. Sixty-six patients (63.4%) were dissatisfied with the effectiveness of the treatment with the medications, and over half (n = 55, 52.9%) reported taking painkillers on a regular basis to manage their chronic headaches.

### 3.4. Correlation between HIT-6 and MIDAS in Subgroups and Clinical Variables

Fifty-seven patients (54.8%) had a severe disability according to the MIDAS score, in contrast to 36 patients (35.3%) based on the HIT-6 score (Table S1). When comparing the little–mild (n = 58) and moderate–severe (n = 44) HIT-6 score groups, the only significant difference was a longer duration since diagnosis in the former group (9.04 ± 7.67 vs. 5.76 ± 4.89 years, *p* = 0.023) (Table S2 Supplemental Materials). 

Patients with higher MIDAS disability scores (grades 3–4, n = 68), in comparison with those with lower disability scores (grades 1–2, n = 36), were younger (32.41 ± 8.93 vs. 41.47 ± 14.36, *p* < 0.001) with a significantly shorter average time since diagnosis (5.93 ± 5.27 vs. 11.73 ± 8.47 years, *p* < 0.001) (Table 2). Additionally, the FARB score was significantly lower (indicating a higher severity of narrowing in the transverse-sigmoid junction) in the high-severity MIDAS group (1.28 ± 1.82 vs. 2.87 ± 2.3, *p* = 0.02). 

Notably, a significant correlation (χ^2^ = 57.32, *p* < 0.001) was found between a high HIT score and a high MIDAS score, suggesting similar directionality. A logistic regression model was used to find predictors of a higher MIDAS score (Table 3).

A negative trend was found for a higher FARB score (OR 0.78, 95% CI 0.57–1.12, *p* = 0.12). Additionally, a higher age showed a negative trend for a higher MIDAS score (OR 0.92, 95% CI 0.84–1.02, *p* = 0.13) while the time since diagnosis did not show any trend (*p* = 0.93). Fifty-seven patients were classified as severe according to the MIDAS scale, with different scores. A Pearson correlation found a tendency for an inverse correlation between the FARB severity score and the MIDAS score (−0.193, *p* = 0.15). A post hoc analysis of patients with severe MIDAS scores, comparing the 10 patients with the highest MIDAS score with the remainder of the subgroup (n = 47), found that they differed only in terms of the FARB score (1.6 ± 1.1 vs. 2.7 ± 2.4, *p* = 0.041) (Table 4).

## 4. Discussion

In the current study, we found that headaches persist and quality of life is impaired in ophthalmologically stable female IIH patients. We focused our study on this subpopulation of females as they comprise the majority of IIH patients, and this evaluation might therefore provide further insights into the quality of the current standard of medical treatment for IIH. Our findings are supported by existing studies that similarly report persistent headaches in a significant percentage of IIH patients despite medical treatment [4,6,9,18,19].

Witry et al. [13] reported a higher ratio of patients (82%) suffering a severe impact of headaches on their daily life (HIT-6 ≥ 60) compared with our study (35.3%). We may attribute this high incidence to the different composition of the study populations. Our study excluded patients who were within the first 6 months after an IIH diagnosis, while they were included in the study by Witry et al. [13]. Moreover, similar results were reported by Xu et al. [6], who showed that almost half (48.8%) of the medically treated patients were still suffering from chronic headaches after an average follow-up period of 2.8 years. A 9-year observational study by Thaller et al. [9] also found a high headache burden. They found that the HIT-6 score was mildly affected by the time interval since diagnosis, and stepwise regression analysis showed that the only factors affecting long-term headache frequency were the occurrence of daily headache at diagnosis and a personal migraine history. Disease duration, change in BMI and family history of migraine were not significantly influential. 

Similar to the study by Thaller et al., the multivariate logistic regression analysis in our study did not show that the time interval since IIH diagnosis had any impact on the MIDAS severity (*p* = 0.93). However, the multivariate analysis in our study showed a clear trend for younger age as a predictor of a moderate–severe MIDAS score (OR 1.09, *p* = 0.13). Possible etiologies may include age-related hormonal changes [20] and structural changes. 

Our multivariate analysis found an additional interesting trend for a lower FARB score as a predictor of moderate–severe MIDAS (OR 1.3, *p* = 0.12). The 10 patients with the highest MIDAS scores were found to have the lowest FARB scores. Previous studies suggested that transverse sinus stenosis with a significant pressure gradient increases cerebral venous pressure, impairs CSF resorption in the venous system, and thereby increases intracranial (CSF) pressure, aggravating the symptoms of IIH [8,21]. To the best of our knowledge, this is the first study incorporating neuroradiology markers into the assessment of medically stable IIH patients’ symptoms and QOL.

Although all the participants experienced complete resolution of their papilledema and did not suffer from a permanent and significant visual field loss with standard medical treatment, 75% experienced significant disability and very poor PROMs. Moreover, 15.7% of the study participants were formally diagnosed with depression or anxiety. Multiple studies have also reported high rates of anxiety and depression in chronic IIH patients, as well as significant disability and poor quality of life (QoL) [6,13,22,23,24,25,26]. Other works found cognitive impairment in multiple domains (e.g., impaired networks (executive function) and sustained attention) in female adult IIH patients compared with controls [16]. Biousse and Newman showed the multidisciplinary manifestations of IIH, which results in visual loss, chronic headaches, chronic tinnitus, depression, and even cognitive impairment, with decreased quality of life and chronic disability being associated with multiple hospital admissions. They concluded that there is a pressing need for a better understanding and improved management of IIH to limit the inevitable burden on healthcare systems around the world. The authors suggested a multidisciplinary, holistic approach to the treatment of IIH, addressing all aspects of the expanding spectrum of IIH and targeting not only direct treatment of intracranial hypertension and papilledema but also aggressive management of headache, CSF leak repair, symptomatic relief, improvement of patient quality of life, and sustained weight loss.

In this cohort, we noticed an improvement in disease symptoms over time, possibly due to age-related changes. Part of the improvement seen in the older IIH patients may have been influenced by the reduction in migraine severity among menopausal women. Our study did not differentiate between headache types, and we therefore could not fully investigate that possibility. Nevertheless, even 10 years after diagnosis, eight participants (7.6% of the entire cohort) had moderate-to-severe MIDAS scores. Our study adds to the current literature supporting the idea that IIH remains chronic in a significant number of patients and that the currently available medications mainly target the ophthalmologic aspect of the disease. From the patients’ perspective, despite the improvement of ophthalmologic symptoms, almost half (47.8%) of our study participants felt discouraged by the ability of medications to treat their illness.

Most of our study cohort was treated with acetazolamide (87%), and the remaining participants were treated with topiramate. In total, 16 of 104 (15.38%) patients reported severe adverse effects attributed to the medications they were given. Severe adverse effects, mainly related to acetazolamide, are well described in the literature [4,6,13,19,27]. Xu et al. [6] reported that during a 1-year follow-up period, 34.2% of the patients stopped the usage of acetazolamide, and 36.4% stopped topiramate due to adverse events. The rate of adherence to acetazolamide during 6 months of the NORDIC trial [7] was 89%, which is better than that reported by Xu et al. [6]. but still reflects difficulties in coping with long-term drug treatment.

In our study, 55/104 (52.9%) patients reported taking painkillers on a routine basis. The routine use of painkillers leading to a degree of overuse has been described in multiple studies. Similar results were presented in a review study by Mollan et al. [23] showing the regular use of analgesic medications in up to half of IIH patients, depending on the study.

As in prior studies, no correlation was found in our study between the severity of pain or disability and the BMI, opening pressure on lumbar puncture, medical background, or papilledema at diagnosis [18,28]. We did not identify any radiological findings that could be used as potential predictors of disability or headache severity, apart from the FARB score. Additionally, we did not find any other studies in the literature that evaluated similar radiological findings as potential predictors for comparison purposes.

Our study demonstrated significant disability (MIDAS), headache (HIT-6), and PROMs in chronic IIH patients treated with medications, even years post-diagnosis. Different studies that investigated the HIT-6 and MIDAS scores, as well as other scores related to QoL, also showed a significant disease burden. Currently, there are no disease-specific validated tools to evaluate QoL and other disease-related symptoms in IIH patients [22]. Until such a tool is available or a large multicenter study is performed, it will be difficult to assess the extent of the disease burden in IIH and hence the efficacy of the current standard treatment.

The currently available medications for the treatment of IIH have not been proven to be efficient for all disease aspects in longitudinal studies. Many studies indicated a high rate of significant adverse effects, which may affect the treatment compliance and may worsen the QoL. We found lower HIT-6 and MIDAS scores in patients diagnosed a relatively long time prior to study participation.

Our study has several limitations. The aim of our study was to evaluate the long-term headache and functional outcomes in ophthalmologically resolved women with IIH. Unfortunately, we did not find well-validated scales for this evaluation in IIH patients. As a result, we chose to include both the MIDAS and HIT-6 in our questionnaire, which are scales validated for migraine patients and not for IIH patients. Notably, our sample size was small, with only 104 patients being included. Another important limitation is the retrospective nature of part of the data collection. Moreover, the questionnaire was administered only once to each patient. Finally, our cross-sectional study did not include questions regarding the phenotype of headaches (characteristics of migraine, tension headache, or both). This is important because Thaller et al. found that the two main factors influencing high headache frequency and worse prognosis were a personal migraine history and daily headache at baseline [9]. Prospective studies with larger samples are required to understand treatment outcomes in IIH patients.

## 5. Conclusions

Similar to previous studies, our study using a detailed questionnaire found that IIH symptoms persist, and quality of life is impaired, even years after ophthalmological stability has been achieved in IIH female patients. We found in our multivariate model that a higher FARB score and older age showed a negative trend for higher severity according to the MIDAS scale score. Finally, the 10 patients with the highest MIDAS severity scores differed from the remainder of the patients with severe MIDAS scores only by having lower FARB scores. Thus, we assume that severe IIH symptomology may be impacted by a narrowing of the transverse sigmoid sinus junction. We regard our findings as hypothesis-generating only, and further large scale, consecutive, prospective imaging-based studies are therefore needed to understand the long-standing symptoms of female IIH patients.

## Figures and Tables

**Table 1:** Demographic and Baseline Clinical Characteristics of Study Participants.

Characteristic	Participants (N = 104)
Age, mean (SD), y	35.6 (11.9)
BMI upon diagnosis, mean (SD), kg/m^2^	34.6 (8.3)
Duration of IIH diagnosis, mean (SD), y	7.9 (7.0)
LP opening pressure at diagnosis, mean (SD), mmH_2_O	361.0 (99.4)
Diabetes	3 (2.9)
Chronic hypertension	14 (13.6)
Polycystic ovary	19 (18.4)
Hypothyroidism	9 (8.7)
Hypertriglyceridemia	10 (9.9)
Anemia	30 (29.1)
ADHD	23 (22.3)
Depression/anxiety	16 (15.7)

Abbreviations: ADHD, attention deficit hyperactivity disorder; BMI body mass index; BP, blood pressure; IIH, idiopathic intracranial hypertension; LP, lumbar puncture.

**Table 2:** Comparison between mild- versus high-severity MIDAS score groups.

MIDAS		Little-to-Mild Severity (Grade 1 + Grade 2) N = 36 (34.6%)				Moderate-to-Severe Severity (Grade 3 + Grade 4) N = 68 (65.4%)					
Variable	Subgroup	N	%	Mean	SD	N	%	Mean	SD	F/χ^2^	*p*-Value
Age		36		41.47	14.36	68		32.41	8.93	F = 15.70	**<0.001**
Time since diagnosis		30		11.73	8.47	61		5.93	5.27	F = 16.08	**<0.001**
BMI		33		34.39	8.88	60		34.69	8.02	F = 0.03	0.87
Medical history											
Diabetes		2	5.7			1	1.5			χ^2^ = 0.35	0.55
Hypertension		8	22.9			6	8.8			χ^2^ = 2.77	0.09
Polycystic ovary		8	22.9			11	16.2			χ^2^ = 0.31	0.58
Hypothyroidism		2	5.7			7	10.3			χ^2^ = 0.17	0.68
Hypertriglyceridemia		3	8.6			7	10.6			χ^2^ = 0	1
Diagnosis of anxiety/depression		6	17.1			10	14.9			χ^2^ = 0	1
Radiology											
Empty sella		14	58.3			35	55.6			χ^2^ = 0	1
Slit-like vent		5	20.8			12	19			χ^2^ = 0	1
Flattening of sclera		11	45.8			32	50.8			χ^2^ = 0.03	0.86
Optic nerve sheath dilatation		18	75			46	73			χ^2^ = 0	1
FARB score		25		2.87	2.3	79		1.28	1.82	F = 5.57	**0.02**
Neuro-ophthalmology											
Papilledema at time of diagnosis		32	88.9			61	89.7			χ^2^ = 0	1
Damage to visual fields at time of diagnosis		11	68.8			29	64.4			χ^2^ = 0	1
Functional difficulties				3.74	2.69			5.38	3.17	F = 5.75	**0.018**
HIT		34				68				χ^2^ = 57.32	**<0.001**
	Minimal	24	70.6			3	4.4				
	Moderate to Substantial	10	29.4			29	42.7				

**Table 3:** Multivariate model for prediction of moderate–severe MIDAS score.

Predictors	Odds Ratios	CI	*p*
Age	0.92	0.84–1.02	0.134
Time since diagnosis	1.05	0.84–1.23	0.931
FARB score	0.78	0.57–1.12	0.121

**Table 4:** Sub-analysis of SEVERE MIDAS group.

Subgroup of SEVERE MIDAS	Highest Severity (n = 10)	Remainder of the SEVERE Subgroup (n = 47)	*p*-Value
MIDAS score ± SD	252 ± 61	84 ± 47	<0.001
Age ± SD (years)	35.6 ± 10.4	32 ± 9.2	0.266
Time from Diagnosis ± SD	6.3 ± 6.3	5.6 ± 5.4	0.735
FARB score	1.6 ± 1.1	2.7 ± 2.4	0.041

## Data Availability

The original contributions presented in the study are included in the article; further inquiries can be directed to the corresponding author.

## Supplementary Materials

The following supporting information can be downloaded at: https://www.mdpi.com/article/10.3390/jcm13133971/s1, Table S1: Degree of Disability in the Study Population as Assessed Using the Headache Impact Test (HIT-6) and the Migraine Disability Assessment (MIDAS) Scores; Table S2: Comparison Between Participants by HIT-6 scores (Little to Mild versus Moderate to Severe).
